# Affinity precipitation of human serum albumin using a thermo-response polymer with an L-thyroxin ligand

**DOI:** 10.1186/1472-6750-13-109

**Published:** 2013-12-17

**Authors:** Zhaoyang Ding, Xuejun Cao

**Affiliations:** 1 State Key Laboratory of Bioreactor Engineering, Department of Bioengineering, East China University of Science and Technology, 130 Meilong Rd., Shanghai 200237, China

**Keywords:** Affinity precipitation, L-thyroxin, Thermo-response polymer, Human serum albumin

## Abstract

**Background:**

Affinity precipitation has been reported as a potential technology for the purification of proteins at the early stage of downstream processing. The technology could be achieved using reversible soluble-insoluble polymers coupled with an affinity ligand to purify proteins from large volumes of dilute solution material such as fermentation broths or plasma. In this study, a thermo-response polymer was synthesized using N-methylol acrylamide, N-isopropyl acrylamide and butyl acrylate as monomers. The molecular weight of the polymer measured by the viscosity method was 3.06 × 10^4^ Da and the lower critical solution temperature (LCST) was 28.0°C.The recovery of the polymer above the LCST was over 95.0%. Human serum albumin (HSA) is the most abundant protein in the human serum system, and it has important functions in the human body. High purity HSA is required in pharmaceuticals. Safe and efficient purification is a crucial process during HSA production.

**Results:**

A thermo-response polymer was synthesized and L-thyroxin immobilized on the polymer as an affinity ligand to enable affinity precipitation of HSA. The LCST of the affinity polymer was 31.0°C and the recovery was 99.6% of its original amount after recycling three times. The optimal adsorption condition was 0.02 M Tris–HCl buffer (pH 7.0) and the HSA adsorption capacity was 14.9 mg/g polymer during affinity precipitation. Circular dichroism spectra and a ForteBio Octet system were used to analyze the interactions between the affinity polymer and HSA during adsorption and desorption. The recovery of total HSA by elution with 1.0 mol/L NaSCN was 93.6%. When the affinity polymer was applied to purification of HSA from human serum, HSA could be purified to single-band purity according to SDS-PAGE.

**Conclusion:**

A thermo-response polymer was synthesized and L-thyroxin was attached to the polymer. Affinity precipitation was used to purify HSA from human serum.

## Background

Since the late 1960s, affinity purification methods have been developed and continuously improved. Affinity precipitation was reported as a potential technology for the purification of proteins during early stages of downstream processing [1]. This technology could be achieved by using reversibly soluble-insoluble polymers coupled with an affinity ligand to purify proteins from large volumes of dilute solution material, such as fermentation broths or plasma. The applications of the technology depend upon the design of efficient synthetic soluble-insoluble polymers, such as pH-, temperature- and light-response polymers. Zhou *et al*. [2] purified lipase using a thermo-response polymer with hydrophobic butyl groups as a ligand. Chen and Hoffman [3] synthesized a copolymer of N-isopropyl acrylamide and N-acryloxysuccinimide and immobilized Protein A on this copolymer, which was used to purify IgG by thermo-precipitation. Ling and Zhu [4] purified BSA using a thermo-sensitive copolymer consisting of N-vinyl-2-caprolactam (NVCL) and methacrylic acid, with copper as the ligand. In our group, a thermo-response polymer was synthesized using N-methylol acrylamide (N-MAM), N-isopropyl acrylamide (NIPA) and butyl acrylate (BA) as monomers [5], and the polymer was applied to purify lysozyme with an immobilized Cibacron Blue F3GA ligand.

Human Serum Albumin (HSA), the most abundant protein in human plasma, has been one of the most extensively studied proteins over several decades [6,7]. It is synthesized in the liver and presents in the blood with a concentration around 40 mg/ml. HSA is the major transport protein in plasma, involved in the distribution and metabolism of many biologically active compounds such as fatty acids, amino acids, natural products and drugs because of the high affinity between HSA and these biological substances [8]. HSA is therefore widely used in pharmaceuticals. Plasma fractionation [9], aqueous two-phase systems [10], affinity beads [11], cation exchange method [12] and affinity membranes [13], among other methods, have been used to isolate albumin from blood plasma or serum. Affinity precipitation can reduce the volume of crude material significantly and improve purification efficiency in the early stage of protein purification. Dyes [14-17] and metal ions [18-20] are generally used as the affinity ligand to purify HSA.

In this study, a thermo-response copolymer, P_NBN_, was synthesized using NIPA, BA and N-MAM, and then L-thyroxin was coupled to the polymer to obtain an affinity matrix, P_NBN_-T. The thermo-response polymer with the L-thyroxin ligand was used for HSA purification by affinity precipitation. L-thyroxin is a synthetic form of a hormone normally secreted by the follicular cells of the thyroid gland and is typically used as a drug to treat hypothyroidism, transported in human serum [21]. Compared with dye or metal ion ligands, it is a relatively safe affinity ligand. As far as we know, the polymer safety is unlikely to be problematic, for the following reasons. First, these monomers are widely used in polymers or copolymers that have been applied for several decades so their safety has been confirmed many times. Moreover, the recovery of P_NBN_-T is so high that there are only trace amounts of the polymer existing in the purified materials and this could be removed in the next purification step.

According to Petitpas and Petersen [22], there are structural interactions between L-thyroxin (PDB ID code 1HK1) and HSA. The phenolic hydroxyl of L-thyroxin contributes specific hydrogen bond interactions with the side chains of Y150 and R257 on HSA. The outer ring of L-thyroxin allows the phenolic hydroxyl to form hydrogen bonds with the side chains of Y411 and S489 on HSA, while the inner ring forms van der Waals contact with the side chains of Q390, N391, L394, A406, and R410. The L-thyroxin carboxylate group forms a hydrogen bond to a water molecule stabilized by D301 from a symmetry-related HSA molecule. We thus speculated that L-thyroxin could be used as a novel affinity ligand for HSA purification.

In this study, L-thyroxin was immobilized as an affinity ligand on a thermo-response polymer comprising NIPA, BA and N-MAM monomers to form an affinity polymer, which was used for HSA purification by affinity precipitation. The adsorption to and elution of HSA from the affinity polymer were investigated and several interesting results were obtained. This paper is the first to report HSA purification by affinity precipitation with a thermo-response copolymer with immobilized L-thyroxin as a novel ligand. Using this system, HSA was purified to high purity in a single step.

## Results and discussion

### Synthesis of P_NBN_

A thermo-response polymer, P_NBN_, was randomly copolymerized using NIPA, BA and N-MAM as monomers. Among the three monomers, NIPA provides thermo-responsiveness, the hydroxyl groups on N-MAM can be used to immobilize a hydrophobic ligand, and BA is used to control hydrophobicity. Considering the recovery and thermo-response character of the polymer and biomolecular stability, NIPA (7.77 mmol/g), N-MAM (0.44 mmol/g) and BA (0.49 mmol/g) were selected as the final ratio. The molecular weight of the polymer measured by gel permeation chromatography (GPC) was 6.52 × 10^4^ Da. The lower critical solution temperature (LCST) and recovery of corresponding polymer were 31.0°C and 99.6% of its initial amount after recycling three times.

### Immobilization of L-thryoxin on P_NBN_

**Figure 1:**
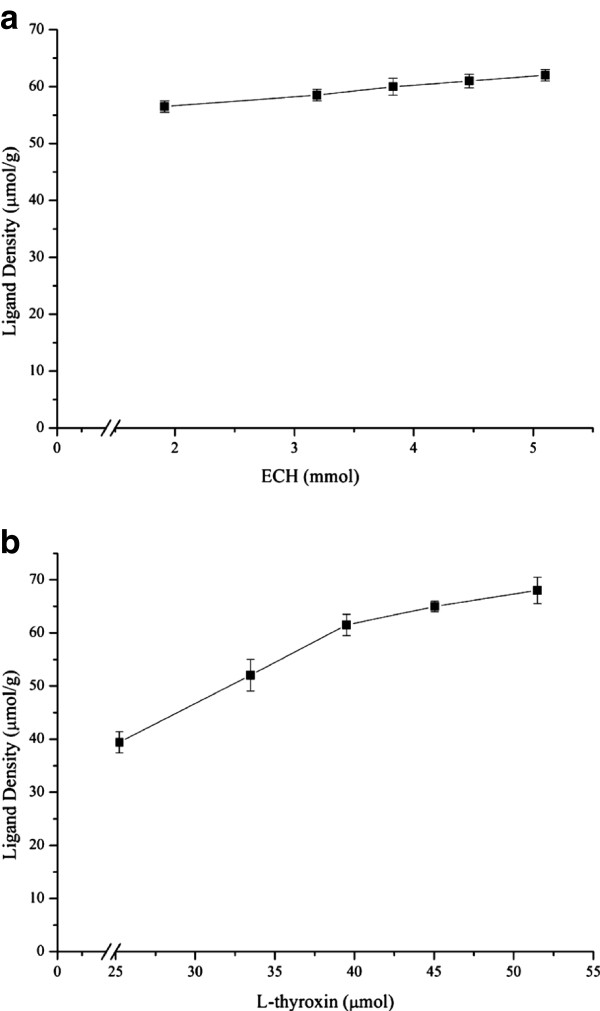
**The results of the Immobilization of L-thryoxin on PNBN. a** Relationship between initial amount of epichlorohydrin in reaction solution and ligand densities on the polymer with 40.0 μmol initial L-thyroxin. **b**. Relationship between initial amount of L-thyroxin in reaction solution and ligand densities on the polymer with 3.0 mmol initial epichlorohydrin.

The ligand and copolymer were connected by an epoxy group from epichlorohydrin (ECH), while its hydrocarbon chain, with three carbon atoms, acted as a spacer between the polymer and the ligand. This spacer was expected to increase contact between the immobilized ligand and HSA. L-thyroxin was activated with excess ECH and then coupled to the P_NBN_ to create P_NBN_-T. Various ligand densities were obtained by changing the initial amount of L-thyroxin, as presented in Figure 1.

When the initial amount of ECH was varied from 1.91 to 5.10 mmol, the densities of the ligand immobilized onto the copolymer increased from 56.10 to 62.35 μmol/g. The ligand density increased by less than 10% with a 2.5 fold increase in ECH, which indicates that the amount of ECH used did not markedly affect ligand density. The reason for this may be that the polymer could only bind to a limited amount of ECH. Figure 1(b) shows that the ligand density underwent a clear increment from 39.10 to 66.30 μmol/g with an increasing initial amount of L-thyroxin. At the same time, the yield of activated L-thyroxin after conjugation to the polymer decreased from 70.18% to 50.54%. The desired ligand density could thus be controlled according to these results.

### Recovery and LCST of P_NBN_-T

Recovery is the most important parameter during affinity precipitation by reversible soluble-insoluble polymers. Measurements of LCST and recovery of P_NBN_-T were performed five times. NaCl addition can increase the recovery of thermo-response copolymers [18] so NaCl was also added to a final concentration of 0.5 mol/L. The maximal recovery achieved was 99.6% and the LCST was 31.0°C.

### Adsorption of HSA

#### Adsorption kinetics curve

**Figure 2:**
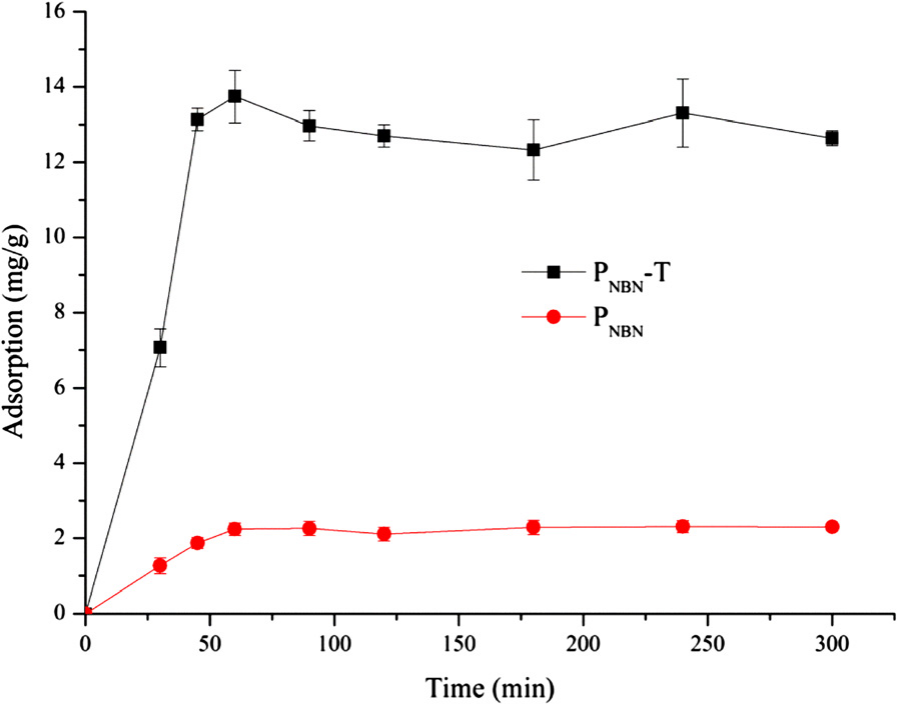
**The effect of reaction time of HSA adsorption on P**_**NBN **_**and P**_**NBN**_**-T.** Initial concentration of HSA 1.0 mg/ml, temperature 25.0°C, pH 7.0, affinity ligand density of P_NBN_-T 60.0 μmol/g and 0.1 g polymer.

As shown in Figure 2, the adsorption capacity of HSA on P_NBN_ increased with time, reaching equilibrium at 30 min, after which time the adsorption capacity was 2.3 mg/g. A relatively fast adsorption of HSA to P_NBN_-T was observed at the beginning of the adsorption process, and adsorption equilibrium was achieved gradually over about 1.0 h. The adsorption capacity was 13.6 mg/g polymer. The polymer P_NBN_ contained hydrophobic groups such as butyl, thus, to some degree, it was hydrophobic. A low amount of HSA was adsorbed on the polymer P_NBN_ via hydrophobic interaction. Compared with the control polymer P_NBN_, the markedly enhanced HSA adsorption capacity on P_NBN_-T can be attributed to the presence of ligand molecules.

As shown in the literature [23], the molecular weight of HSA is about 67,000 Da so the adsorption capacity was equivalent to 0.21 μmol/g affinity polymer when the adsorption reached equilibrium. Compared with the ligand density of 60.0 μmol/g affinity polymer, this is apparently a relatively low binding efficiency. It is probably that the affinity interaction between HSA and L-thyroxin occurs via multi-site binding because it is difficult to understand the low binding efficiency if only mono-site binding occurs. Further study into this aspect should be conducted in the future. The steric hindrance caused by the large size of HSA is also a feasible reason for the low apparent binding efficiency. In subsequent experiments, the reaction time was controlled at 120.0 min to ensure adsorption.

#### Effect of ligand density, pH and ionic strength

**Figure 3:**
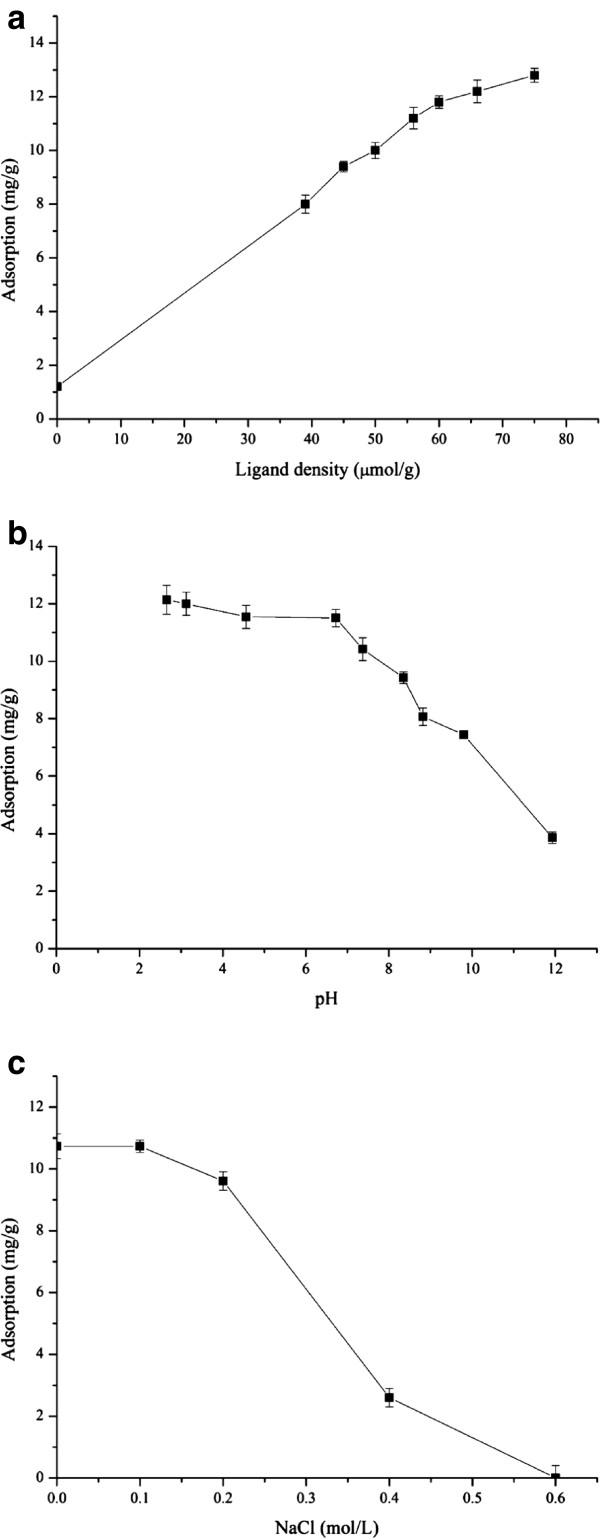
**Different effects of the adsorption capacity. a** Effect of ligand densities of PNBN-T on binding of HSA. Initial concentration of HSA 1.0 mg/ml, temperature 25.0°C, pH 7.0 and 0.1 g polymer. **b.** Effect of pH on binding of HSA onto PNBN-T. Initial concentration of HSA 1.0 mg/ml, temperature 25.0°C, affinity ligand density 60.0 μmol/g and 0.1 g polymer. **c.** Effect of ionic strength on binding of HSA onto PNBN-T. Initial concentration of HSA 1.0 mg/ml, temperature 25.0°C, pH 7.0, affinity ligand density 60.0 μmol/g and 0.1 g polymer.

As shown in Figure 3(a), higher adsorption capacities of HSA were observed at higher ligand densities on the polymer. The adsorption capacity for HSA increased proportionally with ligand densities on the polymer below 60.0 μmol/g affinity polymer. At higher ligand densities, however, the adsorption capacity of HSA increased only gradually. It is possible that this situation arose because of the large amount of HSA adsorbed onto the polymer, which resulted in steric effects such as blockage of binding sites. The ligand density on the polymer applied in subsequent experiments was 60.0 μmol/g affinity polymers.

The pH of the solution has an important effect on the adsorption equilibrium of HSA. In this study, HSA adsorption capacity was slightly decreased from pH 3.0 to 7.0. With an increase in pH, the adsorption capacity decreased sharply, as shown in Figure 3(b). The reason for this decrease in capacity is that the HSA molecules become negatively charged at higher pH values (above the isoelectric point), and the ligands on the polymer are also negatively charged. Electrostatic forces dominate at low salt concentrations, explaining the adsorption curve versus pH behaviors described above.

It can be seen in Figure 3(c) that protein adsorption capacity decreased with increasing ionic strength. NaCl was used to increase the recovery of the affinity polymer at high ionic strength and under this condition, the presence of NaCl reduced the attraction between the HSA and L-thyroxin molecules on the polymer. In subsequent experiments, NaCl was not used for adsorption of HSA.

#### Adsorption isotherm

**Figure 4:**
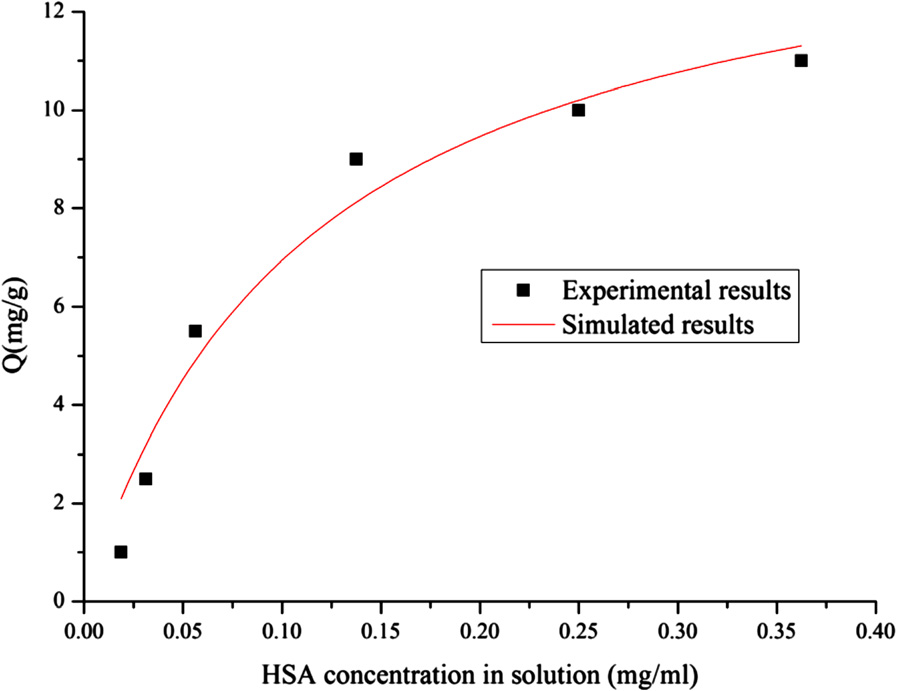
**Adsorption isotherm of HSA binding onto the affinity polymer.** Temperature 25.0°C, pH 7.0, affinity ligand density 60.0 μmol/g and 0.1 g polymer.

Figure 4 shows the adsorption isotherm for HSA binding on the affinity polymer. The curve follows the Langmuir isotherm. The Langmuir model can therefore be applied to fit the experimental data as follows:

(1) $$q=\frac{Q_{m}C}{K_{d}+C}$$

where q is the amount of the adsorbed HSA at equilibrium, C is the equilibrium concentration of unbound HSA in solution, Q_m_ is the maximum capacity of the affinity polymer and K_d_ is the dissociation constant.

Fitting Equation (1) to the experimental data, we obtained Q_m_ as 14.87 mg/g affinity polymer and K_d_ as 0.11 mg/ml. From the molecular weight of HSA, this Q_m_ value is equivalent to 0.22 μmol/g affinity polymer and K_d_ is equivalent to 1.64 μmol/ml. According to Petitpas [22], HSA have several binding sites for the ligand L-thyroxin. As described above and shown in Figure 3(a), the HSA adsorption capacity increased proportionally with ligand densities on polymers below 60.0 μmol/g and increased gradually above that value. This phenomenon may be caused by ligands from different polymer molecules binding onto different sites of each HSA molecule. P_NBN_-T was synthesized for affinity precipitation, which is usually used to purify proteins from large volumes of dilute solution. Both adsorption ratio and capacity are significant factors in this process. The adsorption isotherm was therefore drawn according to these influencing factors. Adsorption follows the Langmuir isotherm and appears to reach Langmuir saturation under the conditions used. Compared with the ligand density of 60.0 μmol/g affinity polymer, adsorption showed a relatively low utilization of ligand. This behavior may be caused by the large size of HSA, resulting in steric hindrance.

### Circular dichroism (CD) measurements

**Figure 5:**
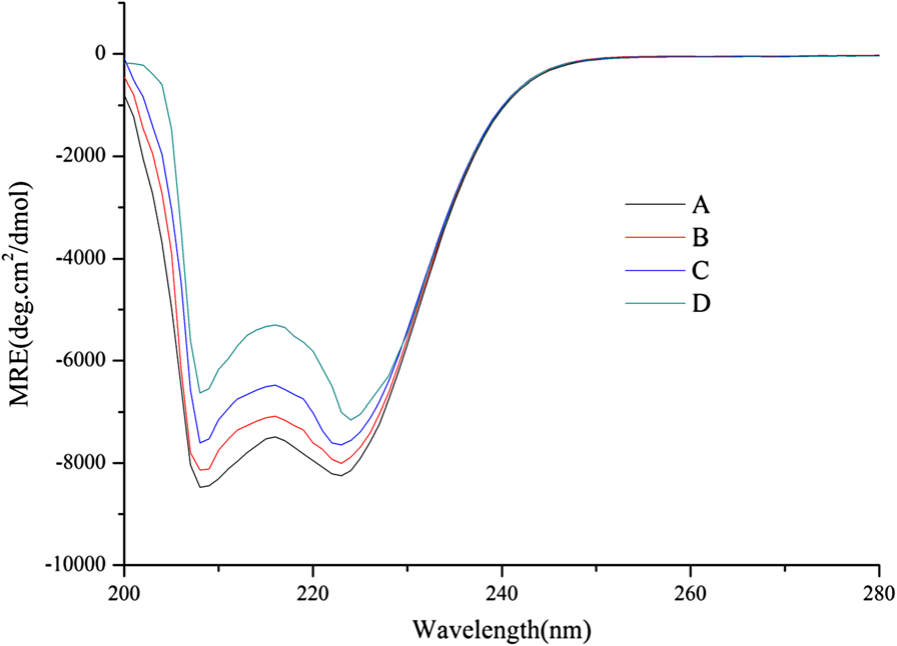
**The CD Spectra of the HSA and L-thyroxin system, obtained in 1.0 μmol/L PBS at pH 7.0 and room temperature.** HSA concentration was fixed at 10.0 μmol/L. In the HSA and L-thyroxin system, L-thyroxin concentrations were 0.0 **(A)**, 20.0 **(B)**, 40.0 **(C)** and 80.0 **(D)** μmol/L.

The CD spectra of HSA without and with L-thyroxin are shown in Figure 5.

The CD Spectra of HSA exhibited two negative bands in the UV region at 208 nm and 222 nm, which are characteristic of a α-helical structure, the content of which can be calculated using Equation (2) [24]:

(2) $$\alpha -\mathrm{helix}\left(\%\right)=\frac{-\mathrm{MR}E_{208\mathrm{nm}}-4,00}{33,00-4,000}\times 100$$

The mean residue ellipticity (MRE) can be calculated by Equation (3) [24]:

(3) $$\mathrm{MR}E_{208\mathrm{nm}}=\frac{\mathrm{observed}\;\mathrm{CD}\left(m\deg\right)}{C_{p}\mathrm{nl}\times 10}$$

where C_p_ is the molar concentration of the protein, n is the number of amino acid residues, and l is the path length. From Equation (2), the α-helix ratio in the secondary structure of HSA was shown to decrease from 15.44% (A) to 14.26% (B), 12.44% (C) or 9.08% (D) as in Figure 5 by adding different concentrations of L-thyroxin. These results indicate that the ligand underwent interactions with HSA, and that increasing the amount of L-thyroxin added caused greater changes in the secondary structure of HSA.

### ForteBio octet system assay

**Figure 6:**
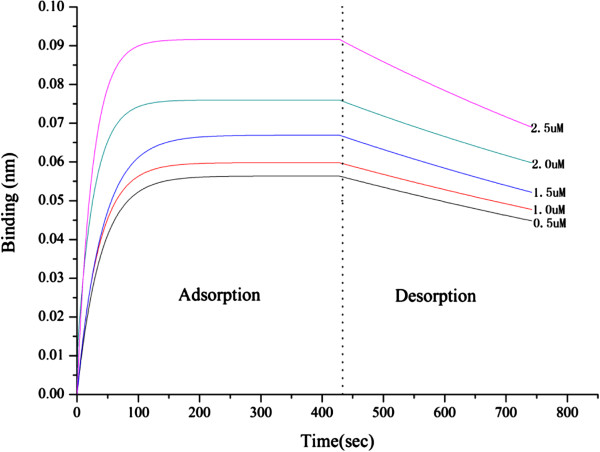
**The vertical and horizontal axes represent the light shift distance (nm) of different concentrations of HSA in the solution and adsorption/desorption time (sec), respectively.** The desorption constant K_d_ (1.42 μmol/L) was calculated from the adsorption and desorption curves of HSA from P_NBN_-T.

The ForteBio Octet system (ForteBio Inc., CA, USA) was used to determine the binding affinities of the ligand to HSA. The dissociation constant K_d_ was calculated by the instrument and calculation software and the K_d_ (1.42 ± 0.45 μmol/L) was in the same range as those obtained from the experiments as in Figure 6.

### Desorption of HSA

#### Efficiency of HSA desorption

Referring to various elution reagents used in other experiments, conditions are shown in Table 1. According to Table 1, about 90.0% of the adsorbed HSA was released from the affinity polymer by using 1.0 mol/L NaSCN as an eluent. However, when other conditions were used for desorption, less than 30.0% of HSA was released. Clearly, NaSCN promotes the dissociation between the protein and the ligand. It was concluded that NaSCN is a suitable eluent.

**Table 1:** Different desorbed conditions

**Desorbed conditions**	**Elution efficiency%**
0.5mol/L NaSCN	89.5
1.0 mol/L NaSCN	93.8
0.1 mol/L EDTA	32.8
pH7.4 Tris–HCl+0.5 mol/L Urea	8.5
pH7.4 Tris–HCl+1.0% Triton 100	10.3
pH7.4 Tris–HCl+20.0% Glycol	24.3
pH9.0 Gly-NaOH	15.5
pH10.0 Gly-NaOH	18.7

### Affinity precipitation of HSA from human serum

Sodium dodecyl sulfate-polyacrylamide gel electrophoresis (SDS-PAGE) and CD measurement were used to analyze the purified HSA.

**Figure 7:**
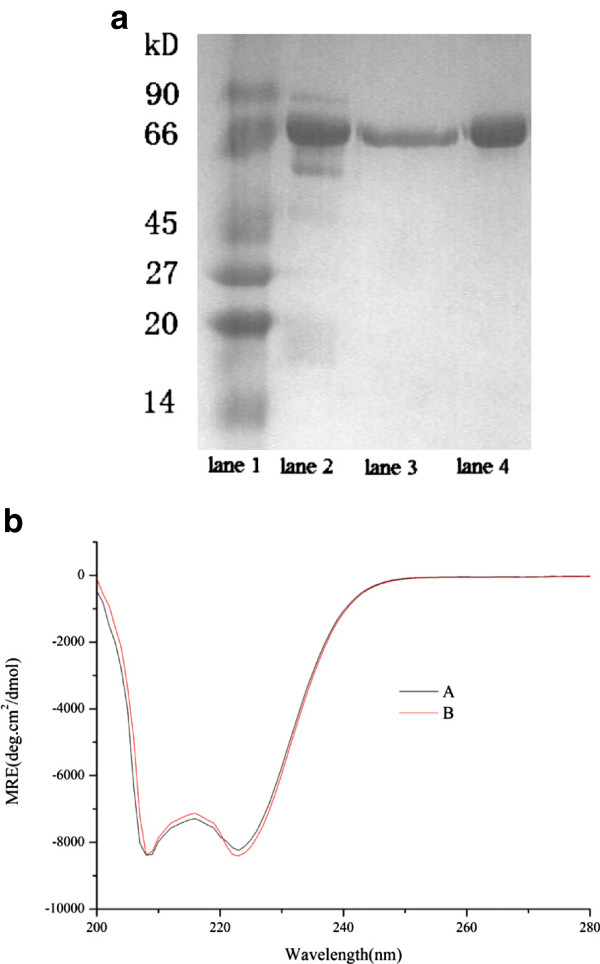
**The SDS-PAGE and CD Spectra results of affinity precipitation. a** Purification effect of HSA with affinity precipitation with 1 mol/L NaSCN as the eluent**.** Lane 1: Protein markers, Lane 2: human serum, Lane 3: HSA after purification, Lane 4: pure HSA. **b** The CD Spectra of HSA, obtained in 1.0 μmol/L PBS at pH 7.0 and room temperature. HSA concentration was fixed at 10.0 μmol/L. **(A)**, pure HSA **(B)**, purified HSA.

Figure 7(a) shows the SDS-PAGE analysis of HSA purified from human serum, showing the molecular weight of HSA was around 67.0 kDa. This indicates that the affinity precipitation was a feasible method for the purification of HSA and electrophoretic purity of HSA can be obtained by a single-step purification. The CD spectra of purified HSA from human serum is shown in Figure 7(b), where both pure HSA and purified HSA exhibited two negative bands in the UV region at 208.0 and 222.0 nm in almost the same range. This result indicates that the secondary structure was unaffected by the process of affinity precipitation. The recovery efficiency from serum was 85.4% in a single step.

### Recycle of P_NBN_-T

To investigate the reusability of the polymer, the adsorption-desorption cycle of HSA was repeated five times using the same polymer sample. The HSA adsorbed onto the polymer was eluted by 1.0 mol/L NaSCN. The results show that the affinity polymers could be repeatedly used for HSA adsorption without any noticeable reduction in recovery. The elution recovery reached 93.8% and the adsorption capacity of the polymer decreased by only 3.8% after five repeated adsorption–desorption cycles. The high recovery from the polymer shows great potential for its application to affinity precipitation.

## Conclusions

A thermo-response polymer P_NBN_ was synthesized, and then the ligand L-thyroxin was coupled to the polymer as an affinity ligand. The LCST of the affinity polymer was 31.0°C and the recovery was 99.6%. An optimal adsorption condition could be identified with a suitable ligand density and pH range. The interactions between HSA and L-thyroxin were confirmed by circular dichroism. A ForteBio Octet system showed the experimental value of K_d_ was consistent with the theoretical value. The total elution recovery of HSA was 93.6% using 1.0 mol/L NaSCN. When the affinity polymer was applied to the purification of HSA from human serum, the purified HSA showed a single band of interest in SDS-PAGE. Thus, this new purification method for HSA could obtain a high purity of HSA in a single step.

## Methods

### Materials

Azobisisobutyronitrile (AIBN), N-isopropyl acrylamide (NIPA), butyl acrylate (BA) and N-methylol acrylamide (N-MAM) were purchased from Sinopharm Chemical Reagent Co., Ltd. (Shanghai, China). Human serum was obtained from Shanghai Yaji Biological Technology Co., Ltd. (Shanghai, China). Pure HSA and L-thyroxin were purchased from Sigma (St. Louis, MO, USA). All other reagents were of reagent grade.

### P_NBN_ polymer polymerization

The synthesis procedure followed Shen’s method [5], developed by our group, with some modifications. A mixture containing specified amounts of the three monomers (NIPA, BA and N-MAM), plus AIBN as a polymerization initiator and ethanol as a solvent were transferred into a flask under a nitrogen atmosphere maintained for 10 min. The reaction was carried out for 24 h at 60°C in a constant temperature bath, and then the ethanol was removed by vacuum distillation. The residue was dissolved in acetone and precipitated by adding an excess of hexane. Finally, the precipitates were collected and dried.

### Immobilization of ligand on P_NBN_ polymer

**Figure 8:**
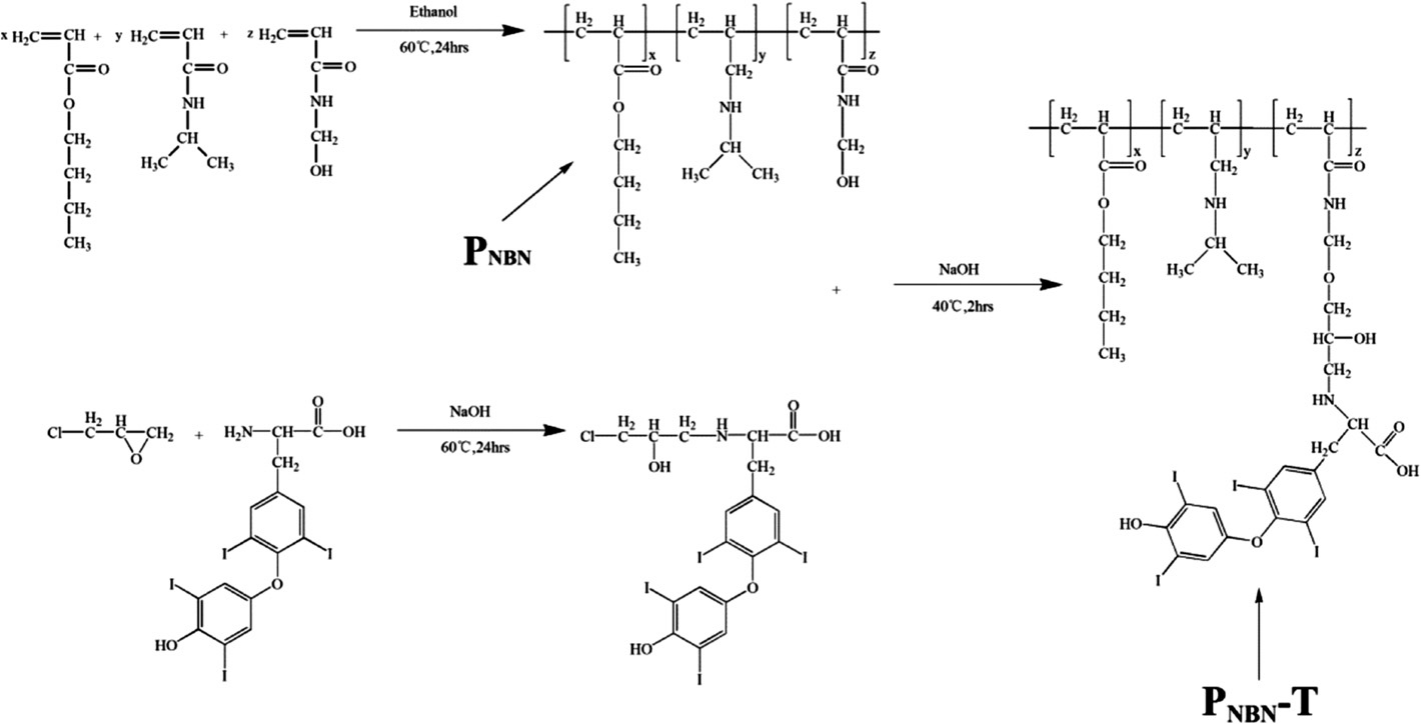
**The reaction formulae for P**_**NBN **_**and the attachment of L-thyroxin.**

Various amounts of L-thyroxin and ECH were dissolved in 45 ml NaOH (1 mol/L) in a conical flask and the activation reaction was carried out for 24 h at 60°C in a constant temperature bath. After that, 5 ml of P_NBN_ (0.1 g/ml) was mixed with the solution and the reaction continued for another 2 h at 40°C in a constant temperature bath. Finally, to remove any unbound ligand, the polymer was precipitated by heating the solution to a temperature above the LCST and it was then washed thoroughly with distilled water. The reaction formulae for P_NBN_ and immobilization of ligand on the P_NBN_ polymer are shown in Figure 8.

### Affinity precipitation of HSA

1) Adsorption

For all samples, unless otherwise specified, Tris–HCl (pH 7.0) buffer was used to dissolve pure HSA. P_NBN_-T was dissolved in an aqueous solution at up to 4.0% (wt/vol). Meanwhile, 2.5 ml HSA solutions of varied concentration were mixed with 2.5 ml of the above P_NBN_-T solution. All samples were kept for 2.0 h at 25.0°C with constant mixing on a rotary shaker in a constant temperature bath. 2.5 ml of NaCl (1.5 mol/L) was added to stimulate the protein-affinity polymer complex to form precipitate at a final NaCl concentration of 0.5 mol/L. Finally, the complex precipitate was collected by centrifugation at 2180 g and 35.0°C for 5 min. The adsorption capacity of P_NBN_-T was investigated by varying the time, pH (3.0–12.0 buffers) and ionic strength (0.0–0.6 mol/l NaCl). The amount of HSA absorbed from the solution was described by mass balance using Equation (4):

(4) $$A=\frac{\left(C_{0}-C\right)\times V}{m}$$

where A is the amount of HSA adsorbed onto P_NBN_-T (mg/g polymer); C_0_ and C are the concentrations of HSA in the initial (before adsorption) and final (after adsorption) solutions, respectively (mg/ml); V is the volume of the solution; and m is the mass of P_NBN_-T (g).

2) Desorption

The collected precipitate was first dissolved in 2.5 ml aqueous solution and mixed with 2.5 ml suitable elution agent. The desorption was carried out in a shaker at 100 rpm for 1 h at 25.0°C in a constant temperature bath. The solution was then heated to 35.0°C to precipitate the polymer and the amount of HSA released within 60.0 min was determined. Desorption recovery was calculated by using Equation (5):

(5) $$\mathrm{De}\left(\%\right)=\frac{D\times 100}{A}$$

where De is the desorbed recovery (%); and D and A are the amounts of albumin desorbed onto and adsorbed from P_NBN_-T, respectively.

3) Affinity precipitation of HSA from human serum

Affinity precipitation of HSA from human serum was carried out by the process described above. The initial concentration of HSA in human serum was determined using an Albumin Reagent Kit within a HITACHI 7060 Automatic Analyzer. Tris–HCl buffer (pH 7.0) was used to dilute the human serum to a final HSA concentration of 5.0 mg/ml. The optimal adsorption and desorption conditions obtained above were applied in this experiment and the result was analyzed by SDS-PAGE. The desorbed recovery of HSA from human serum was determined using Equation (5) and results from the Albumin Reagent Kit and HITACHI 7060 Automatic Analyzer.

4) Recycle of affinity polymer

After HSA desorption experiments were completed, the affinity polymer was recovered and cleaned with 1.0 mol/L NaSCN. The cleaned polymer was reused in the next cycle of purification experiments.

### Analytical methods

1) Testing the LCST

Cloud point measurements were carried out by immersing a test tube containing aqueous P_NBN_ solutions in a water bath heated at a temperature increase rate of 0.5°C /min. A 1 cm sample cell was used and the temperature was set between 22 and 35°C. Concentrations of solutions were in the range of 0.1–10%wt and were analyzed using a Shimadzu UVmini-1240 UV–VIS spectrophotometer. The water-jacketed sample and cell holders were coupled with a THS-10 (Ningbo Tianheng instrument factory, China) programmable circulating bath adjusted at a heating rate of 0.5°C/min. Cloud points were defined as the temperature corresponding to a 10% reduction in the original transmittance of the solution. A similar procedure was followed for the cloud point measurements of P_NBN_-T. LCST was identified as the lowest point in the cloud points curve.

2) Testing the recovery

The recovery of the polymer was calculated as the ratio of the dried weight of the precipitated polymer recovered by heating compared with that of the initial weight.

3) Determination of the ligand density by high-performance liquid chromatography

The high-performance liquid chromatographic (HPLC) system consisted of two LC-20 AD pumps and a SPD-20 A ultraviolet detector (Shimadzu, Japan). All separations were achieved on an analytical reversed-phase ZORBAX C18 column (4.6 × 150 mm) at a flow rate of 1.0 ml/min at room temperature. The UV detector was operated at 212 nm. The two mobile phases used were as follows: phase A: 0.1% phosphoric acid in methanol; phase B: 0.1% phosphoric acid in water.

4) CD spectra assay

CD spectral data can be used to reveal secondary structure information for a protein to monitor conformational changes. This method [25] was used to analyze the interactions between the ligand and HSA.

5) Protein analysis

Protein concentration was determined according to the procedure described by Bradford [26] using bovine serum albumin as a standard.

6) ForteBio Octet system assay

**Figure 9:**
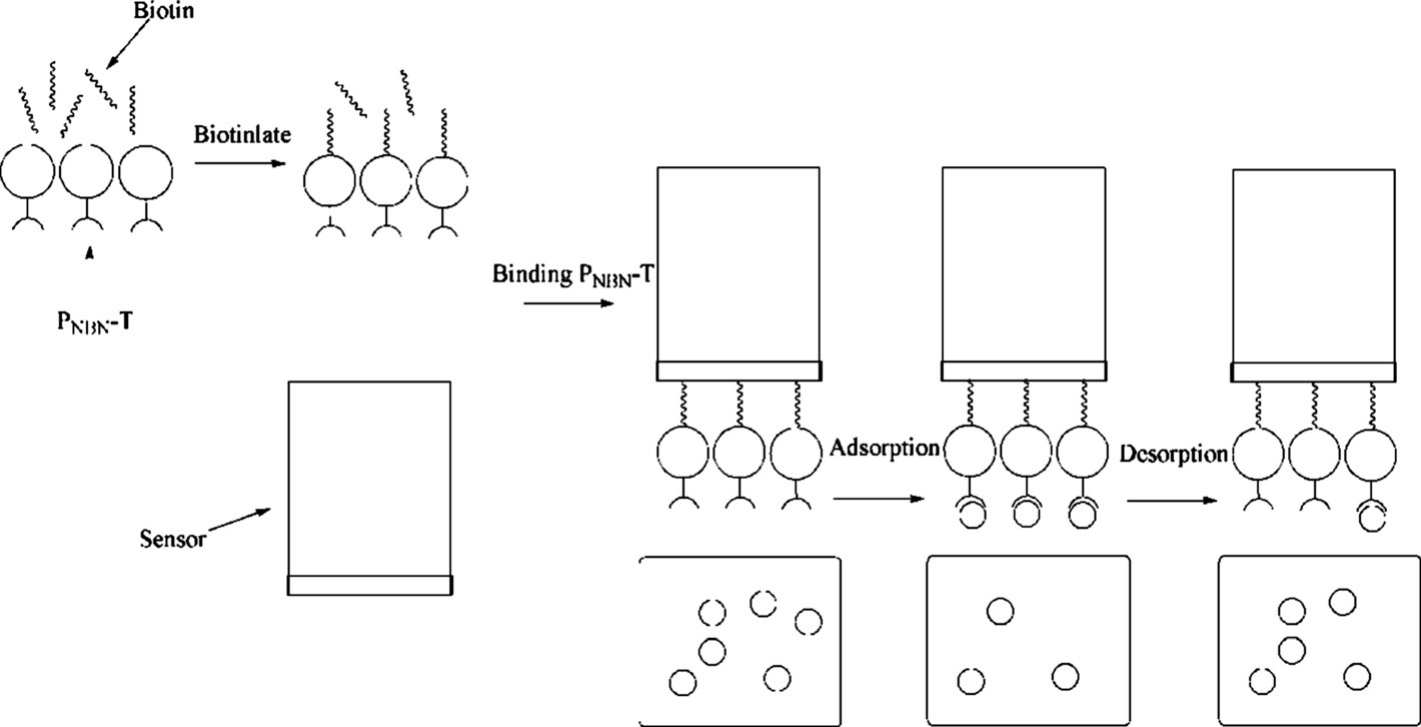
**Immobilization of P**_**NBN**_**-T on biosensors with subsequent HSA binding.** After P_NBN_-T was biotinylated and bound to the biosensors, the kinetics of adsorption and desorption of HSA to immobilized P_NBN_-T were measured.

The interaction between P_NBN_-T and HSA was tested on the ForteBio Octet System (ForteBio Inc., CA, USA). The P_NBN_-T was immobilized onto the biosensors and the processes of adsorption and desorption of HSA molecules monitored in parallel [27]. P_NBN_-T was biotinylated by adding equivalent biotin for 30 min and the unconjugated biotin was then removed using a PD-10 desalting column (Catalog number 17-0851-01, GE Healthcare, USA). The sensors (Super Streptavidin, SSA) were wetted in dialysis buffer for 15 min prior to use. For the binding affinity assay, the sensors were loaded with biotinylated P_NBN_-T for 15 min, then quenched in 10.0 μmol/L biotin for 1 min. Compound HSA was prepared in a serial dilution (0.5, 1.0, 1.5, 2.0 and 2.5 μmol/L). A sensor without loaded biotinylated P_NBN_-T, was used as a control to calibrate baseline drift. The whole experimental procedure (Figure 9) was carried out at room temperature and all tests were repeated in triplicate.

7) SDS-PAGE analysis

SDS-PAGE analysis [28] was performed following the method of Laemmli with a 10.0% separating gel to check the purity of the enzyme sample obtained by affinity precipitation. The gel was stained with 0.25% Coomassie Brilliant Blue R-250.

## Competing interests

The authors declare they have no competing interests.

## Authors’ contributions

ZYD and XJC constructed the idea and methods for this work. ZYD developed the protocol of affinity precipitation and evaluated the data. All authors read and approved the final version of the manuscript.
